# Zooplankton changes during bottom-up and top-down control due to sustainable restoration in a shallow urban lake

**DOI:** 10.1007/s11356-019-05107-z

**Published:** 2019-05-11

**Authors:** Joanna Rosińska, Wanda Romanowicz-Brzozowska, Anna Kozak, Ryszard Gołdyn

**Affiliations:** 1 Department of Environmental Medicine, Faculty of Health Sciences, Poznan University of Medical Sciences, Rokietnicka 8, 60-806 Poznań, Poland; 2 Department of Water Protection, Faculty of Biology, Adam Mickiewicz University, Umultowska 89, 61-614 Poznań, Poland

**Keywords:** Aquatic ecosystem, Biomanipulation, Cladocerans, Phosphorus inactivation, Rotifers

## Abstract

Long-term cyanobacterial blooms and hypereutrophic state have been typical for the shallow, urban Swarzędzkie Lake for many years. Diversion of sewage did not change its trophic status, so restoration began in autumn 2011 using the sustainable approach based on three methods. The aim of the study was to analyse how sustainable restoration affects zooplankton. We hypothesised that bottom-up and top-down methods reconstructed zooplankton composition. Thus, the abundance of large-size cladocerans increased and controlled phytoplankton effectively. The elimination of cyanobacteria bloom, the decrease of rotifer abundance and the twofold increase of filter-feeder effectiveness were observed in summer 2012. However, high phosphorus concentration, lack of regular cyprinid removal and insufficient fish stocking together with high temperature prevented zooplankton from controlling cyanobacteria bloom in summer 2013. Rotifer domination with high trophy species was noted, as before restoration. The number of rotifers decreased in 2014, while crustaceans increased due to the significant decrease of nutrient concentrations and an intensification of biomanipulation treatments. Therefore, summer phytoplankton growth was low, without cyanobacteria dominance. The rebuilding of zooplankton in Swarzędzkie Lake was observed during sustainable restoration. However, the treatments should be intensified when adverse changes were observed to obtain better results for the improvement of water quality.

## Introduction

The imbalance in aquatic ecosystems results from irresponsible human activities and accelerated eutrophication. This phenomenon is observed as cyanobacterial blooms, the disappearance of submerged macrophytes and the general deterioration of water quality (Søndergaard et al. 2007; Wu et al. 2013).

Zooplankton, as one of the major components of the aquatic ecosystem, is affected by biotic and abiotic factors, associated with progressive degradation of the environment. Nutrient concentrations, food quantity and quality (e.g. the presence of cyanobacteria bloom), as well as predation, alter zooplankton successions in water ecosystems (Chen et al. 2012b). The increase of nutrient concentrations is caused by external and internal loading and by the activities of omnivorous fish such as roach *Rutilus rutilus* (L.) and bream *Abramis brama* (L.), which dominate in eutrophic lakes (Bernes et al. 2015) and intensify the release of phosphorus into the water column (Søndergaard et al. 1997; Bernes et al. 2015). These favourable conditions stimulate the growth of phytoplankton (Persson et al. 1999; Kraska et al. 2013) and deeply affect the composition of the zooplankton (Chen et al. 2012b). In such conditions, large-sized cladocerans are preys of planktivorous fish (Kuczyńska-Kippen 2003), which lead to the domination of small-sized species in the planktonic fauna community (Chen et al. 2012b). Therefore, zooplankton grazers cannot control the increasing population of phytoplankton and cyanobacteria bloom (Van Donk et al. 1990; Skov et al. 2003; DeBoom and Wahl 2014). Zooplankton is a good indicator of ecological status and lake trophy (Chen et al. 2012a; Ejsmont-Karabin 2012; Haberman and Haldna 2014). It is the main link between the microbial loop and higher trophic levels (Chen et al. 2012a; Ejsmont-Karabin 2012). Consequently, during restoration treatments, it is necessary to focus not only on nutrient limitation but also on the rebuilding of the zooplankton community by reducing the pressure of zooplanktivorous fish. Then large-sized zooplankton, especially *Daphnia* spp., as efficient filter feeders, can regulate the species composition and productivity of the phytoplankton (Blindow et al. 2000; Kuczyńska-Kippen and Joniak 2010). From the other side, the observation of rotifer community structure dynamics is essential for an understanding of the trophic state changes in aquatic ecosystems (Chen et al. 2012a; Ejsmont-Karabin 2012). This group of zooplankton is more useful because their density and taxonomic composition are affected by food resources and not by predatory interactions; they react rapidly to changing environment conditions (Ejsmont-Karabin 2012).

Many restoration methods, like dredging or some chemical methods, improve water quality quickly (Zhang et al. 2010), but are rather ephemeral. During such treatments, the water ecosystem can change drastically, and for example, some valuable species may be eliminated (Rybak et al. 2017a, b). Therefore, it is necessary to find less aggressive but effective and comprehensive methods of improving the ecological state of lakes (Lu et al. 2016) as required by the Water Framework Directive (European Community 2000). Sustainable restoration is based on several methods applied simultaneously in low intensity, which initiate natural processes for decreasing the trophic state (Gołdyn et al. 2014; Rosińska et al. 2018). The aim of such treatments is to reduce the concentration of nutrients, eliminate frequent cyanobacterial water blooms, rebuild the food web, improve light conditions, inhibit the eutrophication process and finally improve water quality in the lake. A combination of methods, such as moderate oxygenation of water overlying the sediments, an increase of phosphorus inactivation in the water column using low doses of chemical agents (e.g. iron sulphate or magnesium chloride) and an increase of the stock of predatory fish by biomanipulation, brings good effects. It is safer for many organisms inhabiting the lakes because these methods disrupt their mutual relations to a lesser extent. However, the rebuilding of an ecosystem is slower and more gradual (Rosińska et al. 2018). Although the effects of all methods are generally well recognised (Søndergaard et al. 1997, 2007; Scharf 2007), the effects of sustainable restoration based on three methods used simultaneously on zooplankton in shallow lakes are still unclear and not well documented.

The main objective of the study was to analyse the rebuilding of zooplankton structure under the influence of sustainable restoration. The most important were observations conducted during spring (when the growth of crustaceans is significant) and summer (when zooplankton structure reflects trophic changes). We hypothesised that sustainable restoration treatments, based on bottom-up and top-down methods, will cause the reconstruction of zooplankton composition and abundance, the elimination of species characteristic of a hypertrophic state, decrease rotifers and increase the number of large-size cladocerans which would then be able to control phytoplankton and reduce cyanobacterial blooms.

## Materials and methods

### Study area

**Fig. 1:**
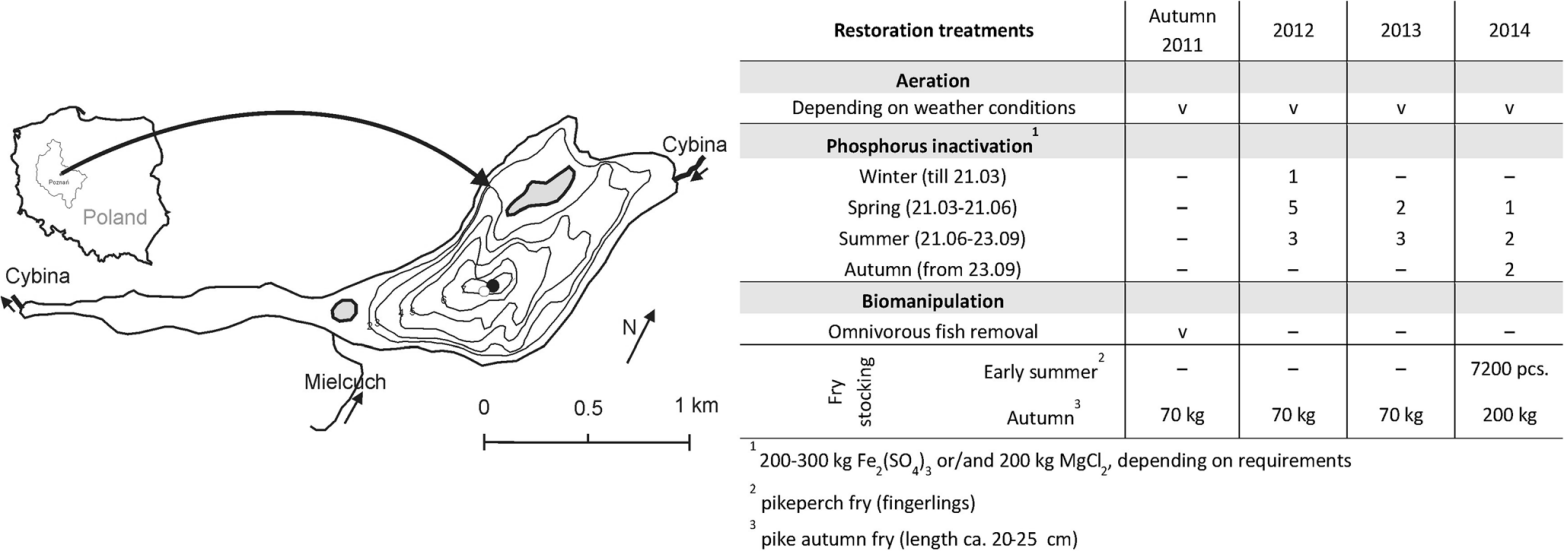
Sampling station (black point) near the aerator (white point) (Kowalczewska-Madura and Gołdyn 2006, changed) and application of sustainable restoration treatments in Swarzędzkie Lake, Poland (Rosińska et al. 2017a, 2018)

The study was conducted in Swarzędzkie Lake, which has been undergoing restoration from autumn 2011 onwards. Swarzędzkie Lake is a relatively shallow (maximum depth 7.2 m; mean depth 2.6 m), through-flow lake, with an area of 93.7 ha (Kowalczewska-Madura and Gołdyn 2006), located on the border of Poznań, West Poland (52°24′49″N 17°03′54″E) (Fig. 1). The lake was classified as a bream–pikeperch type in the 1970s (Rosińska and Gołdyn 2015). It was stocked with European eel *Anguilla anguilla* (L.) and the alien species bighead carp *Hypophthalmichthys nobilis* (Richardson) and silver carp *Hypophthalmichthys molitrix* (Valenciennes) in the 1990s; however, bream, silver bream *Blicca bjoerkna* (L.), roach, as well as bighead carp and silver carp dominated in the catches (Rosińska and Gołdyn 2015). Swarzędzkie Lake has been strongly eutrophic for the last 30 years, the mean Carlson Trophic State Index based on total phosphorus, total nitrogen and chlorophyll *a* concentrations, as well as water transparency, was ca. 70 before restoration (Rosińska et al. 2015), indicating a hypereutrophic state. Favourable conditions for cyanobacterial blooms have been observed for more than 50 years (Kowalczewska-Madura and Gołdyn 2006; Kozak et al. 2014) as a result of the long-term discharge of untreated sewage directly into the lake until 1991 (Gołdyn and Kowalczewska-Madura 2008). Also, high concentrations of nutrients reached the lake with waters of the River Cybina and a small tributary, the Mielcuch Stream, as well as with surface runoff from the adjacent catchment area. Nutrients accumulated in sediments continued to be released to the water column despite sewage diversion and application of other protective measures (Kowalczewska-Madura and Gołdyn 2009).

### Application of sustainable restoration

The main aim of sustainable restoration method applied in the shallow urban Swarzędzkie Lake was to decrease the concentration of nutrients, eliminate of cyanobacterial blooms, reconstruct trophic interactions and increase water transparency. To establish an effective trophic cascade and achieve a long-term improvement of water quality in Swarzędzkie Lake, both bottom-up and top-down approaches have been applied (Rosińska et al. 2017b, 2018). The treatments started in autumn 2011 (Fig. 1). Biomanipulation consisted of catching excess cyprinids, mainly roach, white bream and crucian carp (700 kg in autumn 2011) to reduce the pressure of planktivorous fish on large-sized zooplankton (Kuczyńska-Kippen 2003). There was no presence in the catch of the silver carp and bighead carp, which had been stocked in the 1990s (Rosińska and Gołdyn 2015). Then, during the period of autumn 2011 to autumn 2014, the lake was fourfold stocked with autumn pike fry (length ca. 20–25 cm) and once with early summer pikeperch fry (length ca. 8–10 cm) (Fig. 1) to rebuild the trophic food web (Skov et al. 2002; Bernes et al. 2015) and increase the number of predatory fish (Gophen 1990; Søndergaard et al. 1997; Peretyatko et al. 2012). The pike *Esox lucius* L. reduces the number of planktivorous fish, mainly in the littoral zone, thus ensuring an increase in the size of zooplankton, which in turn decreases the abundance of phytoplankton. The pikeperch *Sander lucioperca* L. is also an efficient predator, which complements the impact of pike within the pelagic zone of eutrophic and hypereutrophic lakes (Wysujack et al. 2002; Jeppesen et al. 2007). The inactivation of phosphorus in the water column consisted of using small doses of iron sulphate (single dose was ca. 2–3 kg Fe_2_(SO_4_)_3_ ha^−1^) and magnesium chloride (single dose was ca. 2 kg MgCl_2_ ha^−1^) applied several times during every year depending on water quality (Fig. 1) (Rosińska et al. 2017a, 2018). The oxygenation of water overlying the bottom sediments using a wind-driven aerator followed, according to the weather conditions (ice cover, a power of wind) (Podsiadłowski et al. 2017). The chemicals caused precipitation of phosphorus from the water column, while increased oxygenation of the sediment–water interphase prevented its internal loading (Dunalska and Wiśniewski 2016; Dondajewska et al. 2018; Siwek et al. 2018). Water quality was analysed during restoration treatments (2012–2014) and compared with results from 2011.

### Field and laboratory research

Zooplankton was sampled monthly from March 2011 to December 2014 (44 months) from the one sampling station at the deepest point of the lake (near the aerator) (Fig. 1). Samples were collected in the depth profile of every 1 m (from the surface to near the bottom—6 m). Ten litres of water sampled with a bathometer was sieved using a plankton net of 40 μm mesh size for every sample of zooplankton (rotifers and crustaceans), and then fixed with Lugol’s solution. Next, qualitative and quantitative analyses of zooplankton (larval stages—nauplii and copepodites were considered together) were carried out with a light microscope, Olympus CX 21 LED. Organisms were identified to species with the identification guides of Radwan (2004) and Rybak and Błędzki (2010). The analyses were based on abundance because it is a more sensitive indicator, especially during periods of changes (Ejsmont-Karabin and Hillbricht-Ilkowska 1994; May and O’Hare 2005). The Shannon index (*H*′) and Pielou index (*J*′) were calculated to evaluate the diversity and species evenness of zooplankton (Sienkiewicz 2010).

To assess zooplankton changes, based on the rotifer trophic state of lake index (TSI_ROT_), the summer samples from epilimnion were analysed (Andronikova 1996; Ejsmont-Karabin 2012). The summer is the best time due to the stability of zooplankton communities, which are under the influence of trophic factors (Ejsmont-Karabin 2012). The epilimnion was determined based on temperature measured in the water column. Then, the mean abundance of particular rotifer species in epilimnion in July and August was calculated. The standard wet weights according to Ejsmont-Karabin (1998) were used to calculate biomass. TSI_ROT_ was determined based on six parameters (Table 1; Ejsmont-Karabin 2012). The crustacean reacts slower and weaker to trophic changes (Ejsmont-Karabin and Karabin 2013). The indices of eutrophication process based on zooplankton abundance according to Andronikova (1996) as well as Haberman and Haldna (2014) were also determined (Table 1).

**Table 1:** The parameters of Trophic State Index based on zooplankton (Ejsmont-Karabin 2012; Ejsmont-Karabin and Karabin 2013), total phosphorus, chlorophyll *a* and water transparency (the results were published—Rosińska et al. 2015) and parameters of trophic state based on zooplankton abundance (^1^Andronikova 1996; ^2^Haberman and Haldna 2014)

	Parameter with formula	B	R1	R2	R3
	Rotifera				
1	Rotifer numbers (*N*, ind. L^−1^) TSI_ROT1_ = 5.38 ln(*N*) + 19.28	66.73	65.98	72.65	64.59
2	Total biomass of rotifer community (*B*, mg w.wt. L^−1^) TSI_ROT2_ = 5.63 ln(*B*) + 64.47	70.35	64.83	73.81	68.55
3	Percentage of bacterivores in total rotifer numbers (BAC, %) TSI_ROT3_ = 0.23 BAC + 44.30	57.56	61.21	61.24	57.56
4	Percentage of the *tecta* form in the population of *Keratella cochlearis* (TECTA, %) TSI_ROT4_ = 0.187 TECTA + 50.38	68.54	66.99	68.57	61.85
5	Ratio of biomass to numbers (*B*/*N*, mg w.wt. ind.^−1^) TSI_ROT5_ = 3.85 (*B*/*N*)^−0.318^	45.63	59.62	53.27	44.51
6	Contribution of species which indicate high trophic state in the indicatory group’s number (IHT, %) TSI_ROT6_ = 0.203 IHT + 40.0	60.18	60.20	60.29	60.06
	TSI_ROT_	61.50	63.14	64.97	59.52
	Trophy based on zooplankton (55–65—eutrophy)	Eutrophy			
	Carlson’s Trophic State Index				
1	TSI_TP_	73.86	75.94	74.47	68.77
2	TSI_Chl_	70.09	62.03	59.09	66.19
3	TSI_SD_	61.35	56.43	57.37	56.79
	TSI mean value	70.80	65.60	65.20	66.40
	Trophy based on physicochemical parameters (50–70—eutrophy)	Hypertrophy	Eutrophy		
	Parameters based on zooplankton abundance				
1	The proportion of Rotifera and Cladocera in total numbers^1^	54	63	245	19
2	The ratio of numbers of Cladocera to numbers of Copepoda—*N*_Clad_/*N*_Cop_^1^	0.29	0.10	0.21	0.42
3	Rotifer abundance (ind. L^−1^)^2^	6761	5882	20,334	4545
4	The percentage share of rotifers in total zooplankton abundance^2^	92.48	84.87	97.68	84.60
5	The ratio of crustaceans abundance to rotifer abundance *N*_Crust_/*N*_Rot_^2^	0.08	0.18	0.02	0.18

The data of zooplankton were also compared with the qualitative and quantitative composition of phytoplankton. Phytoplankton was sampled monthly from March 2011 to December 2014 from the epilimnion layer (from 0 m to 3 m depth) at the same place as zooplankton and analysed with a light microscope, Olympus CX 21 LED. Abundance was determined using a Sedgwick–Rafter chamber with a volume of 0.46 ml and × 400 magnification (Kozak et al. 2014; Rosińska et al. 2018). The organisms were identified to groups with identification guides of Starmach (1989), Huber-Pestalozzi (1983) and Komárek (2005). Temperature, transparency of water (measured as the Secchi disc depth), chlorophyll *a* (acetone method), as well as nitrogen and phosphorus concentrations (Polish Standards, Elbanowska et al. 1999) were also analysed monthly from March 2011 to December 2014, at the deepest place of the lake. The physicochemical and phytoplankton data have already been published in detail (Rosińska et al. 2017a, 2018). However, the means of transparency, chlorophyll *a*, total phosphorus and total nitrogen concentrations from the depth profile for each year were calculated and are given here to show the tendency of changes in water quality during restoration.

### Statistical analyses

The results of zooplankton analyses obtained during sustainable restoration (in vegetative seasons March–September 2012–2014) were compared with the data before the restoration (March–September 2011) and analysed using STATISTICA 13.1 software. The data of zooplankton abundance, Shannon index and species evenness were not normally distributed (Shapiro–Wilk test). Hence, a non-parametric statistical test (Kruskal–Wallis test) was applied. This test was used to verify whether the changes in the abundance, Shannon index (*H*′) and Pielou index (*J*′) of zooplankton which occurred in the depth profile during treatments (2012–2014) were statistically significant in comparison to the period before the restoration measures (2011) (*n* = 164). The significance threshold was *p* < 0.05. The Spearman correlation between abundance of phytoplankton (cyanobacteria and other phytoplankton, i.e. other groups counted together, excluding cyanobacteria) and zooplankton (rotifers, cladocerans, copepods) in epilimnion (from 0 m to 3 m depth) in each year was also tested. The canonical correspondence analysis (CCA) was performed using Canoco for Windows 4.5 software package (Lepš and Šmilauer 2003) to check the dependency of changing abiotic factors with phytoplankton and zooplankton composition before and during restoration treatments (2011–2014). The analyses were done for the period March 2011–November 2014 only for the epilimnion layer (from 0 m to 3 m depth) because it was subject to the most intensive processes related to primary production. Data from the summer (July–August) and from 2013 (year which was distinguished by very high air temperatures) were analysed separately to find how significantly they affect the tested relationship of phyto-, zooplankton versus physical and chemical parameters of water quality.

## Results

### Physicochemical water quality variables

**Fig. 2:**
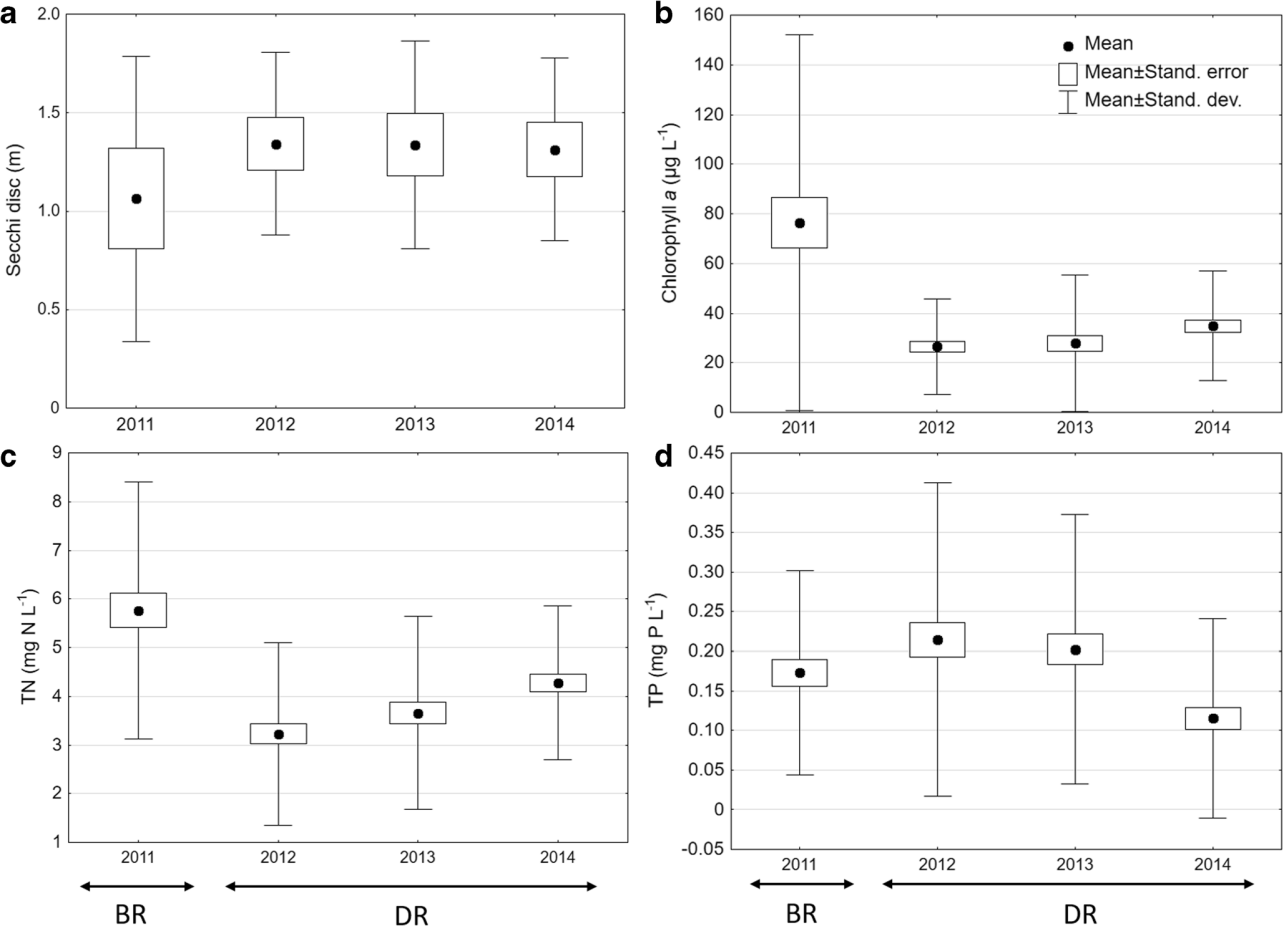
The mean transparency of water (**a**) (Rosińska et al. 2018) and mean concentrations of chlorophyll *a* (**b**), total nitrogen (**c**) and total phosphorus (**d**) from the whole depth profile (from 0 m to 6 m), before (BR) and during restoration (DR) in Swarzędzkie Lake for each year

The average transparency of water in Swarzędzkie Lake was ca. 1.0 m in 2011, before restoration (Fig. 2a). An increasing trend of transparency was observed during restoration treatments and reached ca. 1.3 m (Rosińska et al. 2017a, 2018). The mean concentrations of chlorophyll *a* decreased twofold to fourfold during the restoration (from ca. 80 μg L^−1^ in 2011 to 20–40 μg L^−1^ in 2012–2014) (Fig. 2b). Total nitrogen concentrations decreased significantly in the whole water column every year throughout the restoration process (Fig. 2c), i.e. from 6.0 mg N L^−1^ in 2011 to 4.0 mg N L^−1^ in 2014. The average concentrations of phosphorus were high (especially above the bottom during summer). They fluctuated during the restoration period and finally decreased significantly in 2014, reaching ca. 0.10 mg P L^−1^ (Fig. 2d). The changes in nutrient concentrations and environmental conditions (temperature, length of winter, especially in 2013) affected phytoplankton (Rosińska et al. 2017a, 2018) and, by extension, also zooplankton fluctuation.

### Abundance of zooplankton

**Fig. 3:**
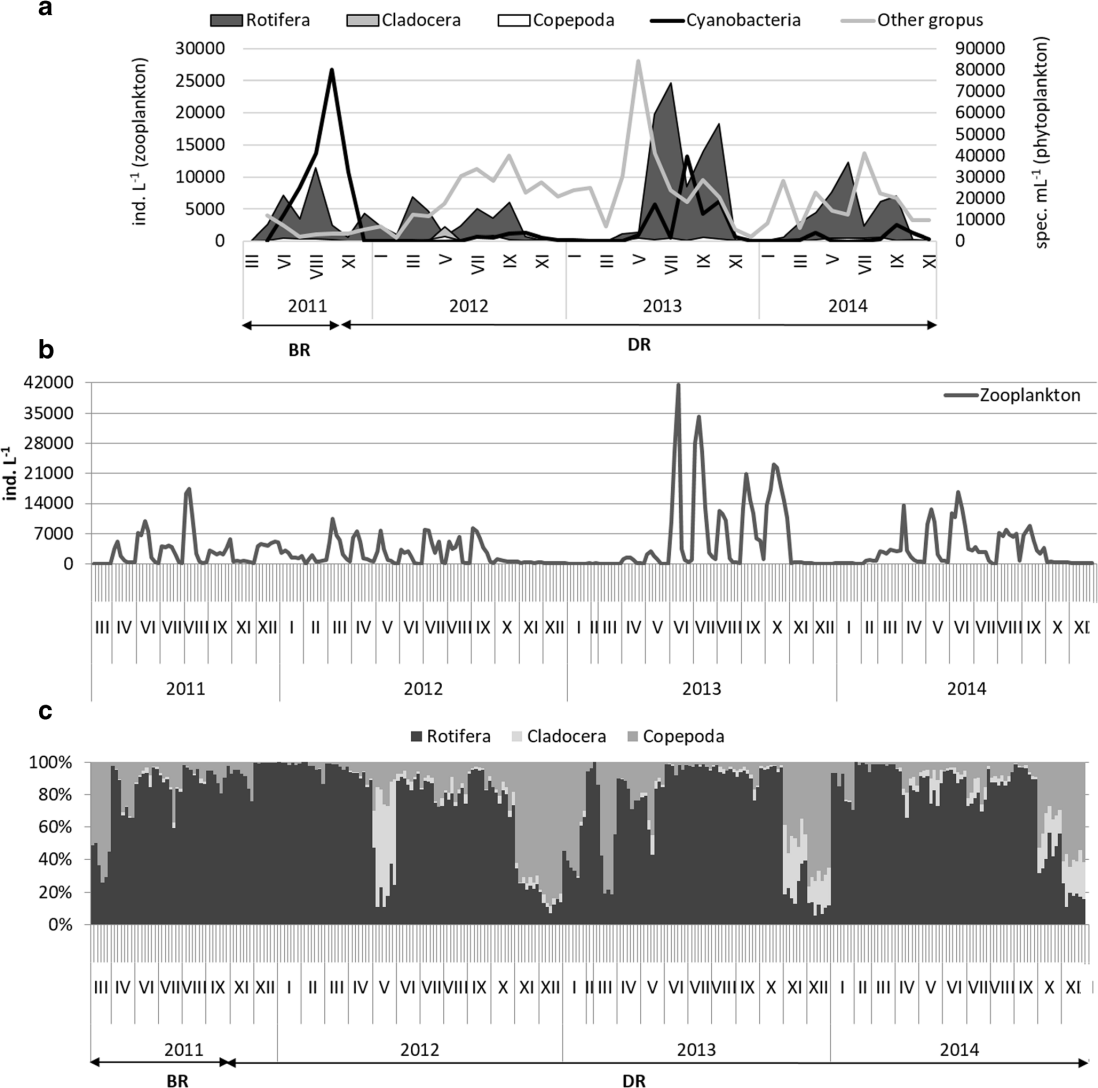
The mean abundance of plankton in the epilimnion layer (from 0 to 3 m depth) (**a**) (Kozak et al. 2014; Rosińska et al. 2018), total abundance of zooplankton (**b**) and percentage share of particular groups of zooplankton (**c**) in the depth profile (from 0 m to 6 m) in Swarzędzkie Lake before (BR) and during restoration (DR)

The highest total abundance of zooplankton occurred in the surface layer (from 0 m to 2 m depth, especially during June–September/October) and reached even 41,619 ind. L^−1^ in June 2013 at a 2 m depth. In deeper water layers, the number did not usually exceed 4000 ind. L^−1^. In general, the abundance of zooplankton was low during late autumn/winter (reached maximum 876 ind. L^−1^ in February 2014), except the winter of 2011/2012, when the abundance reached 1350–5000 ind. L^−1^ (Fig. 3b).

**Fig. 4:**
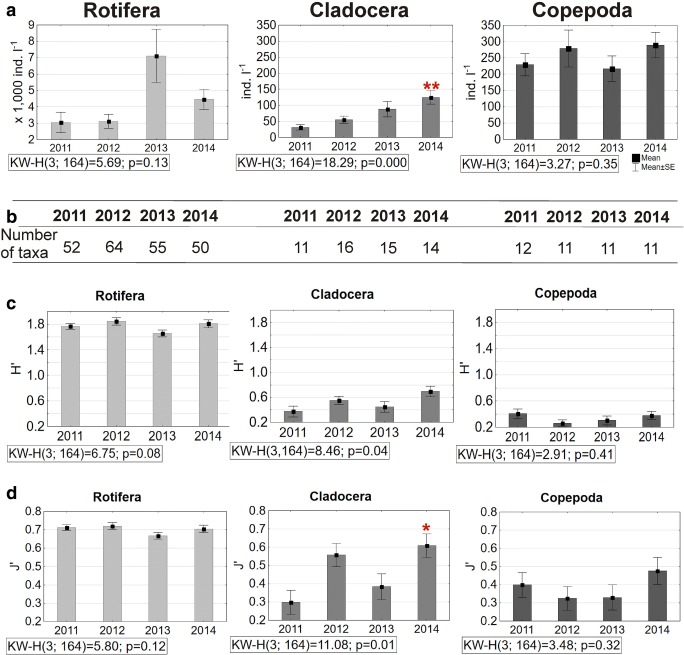
Mean values and standard errors (SE) of abundance (**a**), Shannon index—*H*′ (**c**) and Pielou index—*J*′ (**d**) and number of taxa (**b**) of each group of zooplankton before (2011) and during the restoration (2012–2014) in the water of Swarzędzkie Lake (*p < 0.05, **p < 0.01)

Rotifers were the most numerous and occurred throughout the year. Their number ranged in the depth profile from 16 to 16,800 ind. L^−1^ in 2011, while in 2012 it was lower and ranged from 16 to 10,460 ind. L^−1^. The highest number of rotifers was recorded in 2013 (especially in the period from June to October) and reached its maximum value—40,699 ind. L^−1^ (Fig. 3b, c). In 2014, a lower range of abundance was reported (from 29 to 15,938 ind. L^−1^), similar to 2011. The lowest abundance was noted at a greater depth—at 5 m and 6 m. The changes in rotifer abundance in subsequent years were not statistically significant (Fig. 4a).

Cladocerans were present only in small numbers in summer of 2011 (maximum value was 240 ind. L^−1^ in June at 3 m depth), while during the restoration treatments they occurred more numerously and during most of the year (Fig. 3c). Usually, they were not more numerous in the depth profile than 572 ind. L^−1^, except during May 2012, when there was a peak at a 1 m depth—5655 ind. L^−1^ (the average in the depth profile with the standard error was 1381 ± 761 ind. L^−1^). The abundance of Cladocera was statistically different in 2014 compared to 2011 (*p* < 0.001) and in 2014 versus 2013 (*p* = 0.006) (Fig. 4a).

Copepods were the most numerous from April to November, with a peak in summer (Fig. 3c). They ranged from 9 to 886 ind. L^−1^ in 2011. A notable increase to a maximum 1530 ind. L^−1^ was observed in August 2012, but in 2013 and 2014 the abundance of this group was very similar to that before restoration and did not exceed 900 ind. L^−1^. A lower number was recorded at a greater depth of 5 m and 6 m. The abundance of juvenile copepods (nauplii and copepodites) increased throughout the whole period and even reached 400–500 ind. L^−1^ in summer 2012–2014. Nevertheless, changes in the abundance of copepods were not statistically significant (Fig. 4a).

### The composition of zooplankton and presence of indicator species

Seventy five zooplankton taxa were recorded in 2011, before restoration. Rotifers were the most diverse group (52 taxa), and cladocerans were the least represented (11 taxa) (Fig. 4b). From 75 to 91 zooplankton taxa were observed during the restoration period (2012–2014). Nonetheless, rotifers were still the most diverse group (50–64 taxa). The number of cladoceran species increased and ranged from 14 to 16 (the highest number was in 2012). The number of species of copepods remained at a stable level, approximately 11–12, throughout the study period (Fig. 4b).

The dominant rotifer taxa before and during restoration were *Anuraeopsis fissa* (Gosse), *Brachionus angularis* (Gosse), *Filinia longiseta* (Ehrenberg), *Kellicottia longispina* (Kellicott), *Keratella cochlearis* (Gosse), *K. cochlearis* f. *tecta*, *K. quadrata* (Müller), *Lecane inermis* (Bryce), *Polyarthra dolichoptera* (Idelson), *Pompholyx* sp., *P. sulcata* (Hudson), *Synchaeta* sp. and *Trichocerca rousseleti* (Voigt). Most of these taxa are indicator species for eutrophy (Radwan 2004; Ejsmont-Karabin 2012). During summer, there were also noted two species characteristic for a very high trophy (hypertrophy): *Brachionus diversicornis* (Daddy)—it was present only during restoration period, but its abundance was low and decreased, and *Trichocerca stylata* (Gosse), which was observed only in 2011. Nevertheless, there were also noted the low abundance of two species characteristic for oligo-mesotrophy—*Kellicottia longispina* (Kellicott) and *Polyarthra major* (Burckhardt) (Ejsmont-Karabin and Karabin 2013; Haberman and Haldna 2014). Among cladocerans, *Bosmina longirostris* (Müller)—a species characteristic of eutrophic waters, *Eubosmina coregoni* (Baird) and *Daphnia cucullata* (Sars)—a species of medium size (Rybak and Błędzki 2010) and characteristic for lower trophy (Ejsmont-Karabin and Karabin 2013) dominated in May 2012, autumn 2013 and summer 2014. Other eutrophy species, as *Mesocyclops leuckarti* (Claus), *Thermocyclops oithonoides* (Sars) and *Chydorus sphaericus* (O.F. Müller) (Ejsmont-Karabin and Karabin 2013), although less abundant in summer, also were noted in Swarzędzkie Lake. Copepods, mainly larval stages, i.e. nauplii and copepodites (Cyclopoida), were observed numerously in May, July, August and September or October.

### Zooplankton indices of eutrophication and trophic state

TSI_ROT_ was calculated based on six parameters (Table 1). All species of rotifers, which were indicated by Ejsmont-Karabin (2012) as characteristic for a high trophy and belonged to bacterivorous species, were observed in Swarzędzkie Lake. Their highest abundance was noted during summer 2013 (especially *K. cochlearis* f. *tecta*—the mean was 9405 ind. L^−1^). The indices of eutrophication based on zooplankton abundance (with the exception of two crustacean indices) and all TSI_ROT_ parameters had the highest values during the second year of restoration. However, almost all parameters decreased during the restoration process. Nevertheless, the TSI_ROT_ still indicated eutrophy as before restoration, similarly to Carlson’s Trophic State Index (Table 1).

### Shannon index and Pielou index

The mean year Shannon index for rotifers varied from 1.65 (in 2013) to 1.85 (in 2012), for cladocerans from 0.37 (in 2011) to 0.70 (in 2014) and for copepods from ca. 0.26 (2012) to 0.41 (2011) (Fig. 4c). An upward trend was observed only for cladocerans, but the index changes were not statistically significant. The mean species evenness (Pielou index) was similar for rotifers during the whole study period (0.67–0.72) and varied for cladocerans from 0.30 (in 2011) to 0.61 (in 2014) and for copepods from 0.32 (in 2012) to 0.47 (in 2014) (Fig. 4d). The lowest values for cladocerans were recorded during the cyanobacteria blooms in 2011 and 2013. However, the increase of the *J*′ index was statistically significant in 2014 versus 2011 (*p* = 0.03) (Fig. 4d). Changes for other groups of zooplankton were not statistically significant.

### Plankton interactions

Comparing zooplankton abundance and composition in 2011–2014, the average number of rotifers showed large fluctuations, while cladocerans and copepods displayed a slight upward tendency during treatments (Fig. 3b, c). The dynamics of phytoplankton in Swarzędzkie Lake were closely related to the abundance and taxonomic composition of zooplankton (Fig. 3a). The highest abundance of phytoplankton (cyanobacteria blooms, mainly *Pseudanabaena limnetica* Lemmermann) in the surface water layer stated in 2011 and 2013 was accompanied by a higher abundance of rotifers than in other years (Fig. 3a).

There was an intensive growth of cyanobacteria after the high abundance of rotifers in summer 2011 and 2013. On the other hand, an intensive increase of rotifers was followed by the highest abundance of other groups of phytoplankton (without cyanobacteria) in 2013–2014 (Fig. 3a). A significant correlation between cyanobacteria and all groups of zooplankton was observed in 2013 (with rotifers *ρ* = 0.71, cladocerans *ρ* = 0.60, copepods *ρ* = 0.59; *p* < 0.05). A negative correlation between cyanobacteria and Cladocera was also noted in 2014 (*ρ* = − 0.47; *p* < 0.05). Other phytoplankton (counted together, without cyanobacteria) correlated with Cladocera and Copepoda in 2012 (*ρ* = 0.56 and *ρ* = 0.45, respectively, *p* < 0.05) and with Rotifera and Copepoda in 2013 (*ρ* = 0.48 and *ρ* = 0.49, respectively, *p* < 0.05).

#### Zooplankton versus phytoplankton and physicochemical parameters

**Fig. 5:**
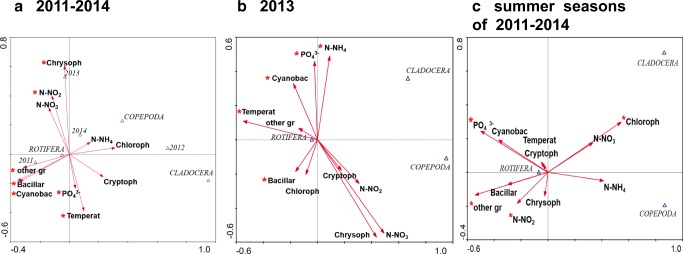
The CCA analysis of environmental variables (Temperat—temperature, PO_4_^3−^—phosphates, N-NH_4_—ammonium nitrogen, N-NO_3_—nitrate nitrogen, N-NO_2_—nitrite nitrogen), compared with zooplankton and phytoplankton (Bacillar—diatoms, Chloroph—chlorophytes, Chrysoph—chrysophytes, Cryptoph—cryptomonads, Cyanobac—cyanobacteria, other gr—rest of phytoplankton) in 2011–2014 (**a**), in 2013 (**b**) and in summer seasons of 2011–2014 (**c**); *p < 0.05

The results of CCA showed that 2011 was dominated by rotifers and cyanobacteria (Fig. 5a). At the beginning of restoration, in 2012, cladocerans and copepods effectively controlled the growth of cyanobacteria. Chrysophytes and chlorophytes dominated in different years of restoration, which were dependent on the nitrate and nitrite concentration in 2013 and ammonium nitrogen in 2012 and 2014, respectively. Rotifers dominated, while crustaceans could not control the proliferation of phytoplankton (Fig. 5a). This resulted from favourable environmental factors, especially in 2013 (Fig. 5b). Cyanobacteria depended on the temperature, which in 2013 was higher than in the remaining years of the study (the mean temperature in June–August was above 25 °C; Rosińska et al. 2017a), and additionally on the concentrations of phosphates and ammonium nitrogen, while chrysophytes, chlorophytes and cryptomonads depended on the concentration of nitrates. These groups of organisms were not directly dependent on crustacean’s abundance (both cladocerans and copepods) (Fig. 5b). In addition, the results of analyses for data from summer periods (Fig. 5c) are largely analogous to results from whole years (Fig. 5a). However, the most important difference concerns the close dependence of chlorophytes on nitrates in summer (Fig. 5c).

## Discussion

Intensive external nutrient loading from different sources, mainly surface runoff and inflow from the River Cybina and Mielcuch Stream, caused strong cyanobacteria bloom in Swarzędzkie Lake (Kowalczewska-Madura and Gołdyn 2006; Kozak et al. 2014). Domination of cyanobacteria indicates the advanced eutrophication of the aquatic ecosystem and disrupts food-web processes (Ger et al. 2014). Therefore, as in many other hypereutrophic lakes, the predation of very abundant cyprinids affected the zooplankton composition intensively (Skov et al. 2002; Adamczuk and Kornijów 2011). Thus, the main purpose of sustainable restoration in Swarzędzkie Lake was to decrease the concentration of nutrients (especially phosphorus) using small doses of chemicals and aeration (bottom-up control) as well as by releasing zooplankton from the food pressure of cyprinids, limiting their numbers by catching and stocking with predators (top-down control). These treatments should limit the abundance of phytoplankton by reducing the availability of nutrients and intensifying zooplankton grazing and as a consequence improve water transparency, as evidenced by Shapiro et al. (1975), Søndergaard et al. (2007) and Bernes et al. (2015).

Zooplankton is a key component of the aquatic environment and is essential to maintaining natural processes in freshwater ecosystems (Sługocki and Czerniawski 2018). Zooplankton indices, based on taxonomic composition, abundance and trophic levels, illustrate the changes (Andronikova 1996; May and O’Hare 2005) taking place during restoration. Previous research conducted in 2000–2002 in Swarzędzkie Lake showed that zooplankton was also dominated by rotifers (Gołdyn and Kowalczewska-Madura 2008), as in 2011, which indicated a high trophic state (Kuczyńska-Kippen and Joniak 2016) and is characteristic for shallow lakes (Sługocki and Czerniawski 2018). The zooplankton composition did not rebuild radically due to restoration. The same species of eutrophic rotifers as well as cladocerans also occurred and dominated in 2012–2014, especially during summer. Nevertheless TSI_ROT_ indicated positive changes. The trophy decreased, although it was still eutrophy. Also, the increase of the diversity index (*H*′) for each group of zooplankton indicated positive biodiversity changes during the restoration. This resulted from the alteration in environmental variables, according to the theory of intermediate disturbance (Reynolds et al. 1993). The Pielou index (*J*′) was high for rotifers during the whole research period, which indicated that variations in the community as a result of the applied treatments were low (Sienkiewicz 2010). An increasing tendency of the values of the evenness index was observed for both cladocerans and copepods, which means that there was an increase in species number, although with no clear dominance of any one of them.

The effect of the applied treatments in Swarzędzkie Lake was well visible during the first year of restoration. An increase of water transparency, a reduction in the abundance and rebuilding of phytoplankton composition along with the disappearance of cyanobacterial bloom were observed (Rosińska et al. 2017a). Such a reaction of the lake ecosystem was a result of primarily bottom-up control, involving inactivation of nutrients in the water column. Nevertheless, the increase of cladocerans in parallel with restoration treatments seemed to be the result of top-down control, including the catching of the cyprinids and stocking with predatory fish in autumn 2011. The high abundance of *D. cucullata* (over 300 ind. L^−1^ at a depth of 1 m in May 2012), the medium-sized species typical mostly for eutrophic lakes and ponds, but an efficient filter feeder, was the key factor in phytoplankton limitation (Mátyás et al. 2004) and evidenced of water quality improvement. An increase of larger cladocerans in late spring 2012 according to the model PEG (Plankton Ecology Group; Sommer et al. 1986) resulted in high grazing pressure on phytoplankton and the effect of the ‘clear-water phase’. A shift from filamentous cyanobacteria (mainly *Pseudanabaena limnetica* with *Aphanizomenon gracile* and *A. flos-aquae* dominated in 2011) to small-sized cells of Chlorophyta was observed (Rosińska et al. 2017a). The increase of cladocerans also indicated predatory fish pressure on planktivorous fish, which resulted in reduced food pressure on zooplankton (Søndergaard et al. 1997; Jeppesen et al. 2002, 2007; Czerniawski et al. 2015). A similar reaction was observed in Lake Zwemlust (Van Donk et al. 1990) and Lake Library (Tüzün and Mason 1996) during the first period of zooplanktivorous fish reduction. An increase in the number of larger rotifers *B. angularis*, *B. calyciflorus*, *Filinia longiseta* and filtering cladocerans *B. longirostris* was also recorded in these lakes (Van Donk et al. 1990) similarly to Swarzędzkie Lake.

A rapid development of small-bodied rotifers takes place in a rapidly changing environment (Ejsmont-Karabin 2012) and their domination is a common phenomenon (Kuczyńska-Kippen 2001; Wilk-Woźniak et al. 2014), especially when high nutrient concentrations and high density of cyprinids occur (Søndergaard et al. 1997; Gołdyn et al. 2010; Chen et al. 2012b; Ger et al. 2016). The increase of primary producers (e.g. cyanobacteria bloom) is observed together with the high abundance of small-sized zooplankton (Langeland 1990; Ger et al. 2014). Such a phenomenon occurred in Swarzędzkie Lake in summer 2013, when the domination of filamentous cyanobacteria (mainly *P. limnetica*) was observed (Rosińska et al. 2017a), and the abundance of rotifers was twofold higher than in the other study periods. The high abundance of *Bosmina* spp. and lower abundance of *Daphnia* spp. in August 2013 indicated the high density of planktivorous fish fry as *Bosmina* is too small to be under fish control (Søndergaard et al. 1997). *B. longirostris* and *Eubosmina coregoni* were not sufficiently effective in controlling phytoplankton and hardly edible filamentous cyanobacteria. Therefore, a water bloom was observed that summer. Numerous rotifers from such genera as *Polyarthra*, *Pompholyx*, *Trichocerca* and *Synchaeta* and domination of *K. cochlearis* f. *tecta* were noted. They feed mainly on detritus and bacterioplankton (Arndt 1993; Radwan 2004), which occurred during the blooms, wherefore they indicate high trophy (Ejsmont-Karabin and Hillbricht-Ilkowska 1994; Ejsmont-Karabin 2012). The low abundance of large herbivorous cladocerans and domination of rotifers in Swarzędzkie Lake in 2013 may have resulted from predation by planktivorous fish (Kozak and Gołdyn 2014). However, the bottom-up process could also have an impact, especially in a shallow eutrophic lake. Cladocerans are more sensitive to Cyanobacteria than copepods and rotifers. Therefore, their density decreased in lakes with cyanobacteria blooms (Ejsmont-Karabin and Karabin 2013). The occurrence of cyanobacteria bloom in that year was probably an effect of several factors. The lower number of chemicals applied within the restoration (twofold less in comparison to 2012, the nutrient concentrations were still high), lack of repeated cyprinid removal and higher temperature (Rosińska et al. 2017a) led to more intensive internal loading (de Senerpont Domis et al. 2013) and resulted in an increase of phytoplankton (mainly inedible cyanobacteria) and rotifer abundance (Haberman et al. 2007; Pociecha and Wilk-Woźniak 2007; Yin et al. 2018). The importance of temperature was also observed by high abundance of thermophilic species during summer, which favour higher trophy, as *K. cochlearis*, *Polyarthra* sp., *A. fissa* and *T. pusilla* (Haberman and Haldna 2014; Yin et al. 2018).

A reduction in the number of large species of crustaceans and the prevalence of smaller cladocerans *B. longirostris* and copepodites (Cyclopoida) were observed in Swarzędzkie Lake not only in 2013 but also partly in 2014. This is a typical symptom of the growing pressure of planktivorous fish (Adamczuk and Kornijów 2011). In addition, the area of the hypereutrophic plant community *Ceratophylletum demersi* decreased during the second and third year of restoration treatments (Rosińska et al. 2017b), which could also have contributed to reducing the success of pike recruitment as a phytocoenotic species (Hilt et al. 2010). Nevertheless, the total abundance of crustaceans again increased (significantly for cladocerans), while rotifers decreased during the third year of restoration. Therefore, it seems that a significant decrease of phosphorus concentration as a result of the restoration, with the simultaneous increase in nitrogen concentration, caused a shift from the growth of cyanobacteria towards other phytoplankton groups, which could be controlled by smaller cladocerans. Intensification of biomanipulation by spring stocking with pikeperch fingerlings (in May 2014) helped limit the pressure of cyprinids on planktonic crustaceans, resulting in improved water quality and reduced phytoplankton growth in summer.

## Conclusions

In summary, the analysis of the composition and abundance of zooplankton and phytoplankton in Swarzędzkie Lake indicated positive effects of sustainable restoration for the reconstruction of lake biocoenosis from hypereutrophic to eutrophic conditions. These effects were already clearly visible in the first year of restoration, probably due to frequently repeated inactivation of phosphorus in the water column, supported by biomanipulation and oxygenation of water overlying the sediments. This contributed to an increase of cladocerans and a decrease of phytoplankton abundance, and the almost complete disappearance of cyanobacteria. The lower number of chemical applications in the following year seemed to be insufficient to maintain good water quality in 2013. The lack of fish removal, the pressure of cyprinid hatching due to the lack of spring stocking of predator fry and the presence of adverse environmental factors (high temperature, internal loading) were too strong, and as a consequence, the abundance of cladocerans decreased, while simultaneously rotifers increased, and a cyanobacterial water bloom appeared as a feedback effect in summer 2013. The gradual decrease of phosphorus concentration and the intensification of biomanipulation due to pikeperch fingerling stocking in the third year of restoration avoided the feedback effect, allowing a clear improvement in water quality, without cyanobacteria dominance.

Using a few methods simultaneously is therefore very important in sustainable restoration as it allows the feedback effect to be overcome and gradual reconstruction of the ecosystem, resulting in improved water quality.
